# BEN domain protein Elba2 can functionally substitute for linker histone H1 in *Drosophila in vivo*

**DOI:** 10.1038/srep34354

**Published:** 2016-09-30

**Authors:** Na Xu, Xingwu Lu, Harsh Kavi, Alexander V. Emelyanov, Travis J. Bernardo, Elena Vershilova, Arthur I. Skoultchi, Dmitry V. Fyodorov

**Affiliations:** 1 Albert Einstein College of Medicine, Department of Cell Biology, Bronx, NY 10461, USA

## Abstract

Metazoan linker histones are essential for development and play crucial roles in organization of chromatin, modification of epigenetic states and regulation of genetic activity. Vertebrates express multiple linker histone H1 isoforms, which may function redundantly. In contrast, H1 isoforms are not present in Dipterans, including *D. melanogaster*, except for an embryo-specific, distantly related dBigH1. Here we show that *Drosophila* BEN domain protein Elba2, which is expressed in early embryos and was hypothesized to have insulator-specific functions, can compensate for the loss of H1 *in vivo*. Although the *Elba2* gene is not essential, its mutation causes a disruption of normal internucleosomal spacing of chromatin and reduced nuclear compaction in syncytial embryos. Elba2 protein is distributed ubiquitously in polytene chromosomes and strongly colocalizes with H1. In H1-depleted animals, ectopic expression of Elba2 rescues the increased lethality and ameliorates abnormalities of chromosome architecture and heterochromatin functions. We also demonstrate that ectopic expression of BigH1 similarly complements the deficiency of H1 protein. Thus, in organisms that do not express redundant H1 isoforms, the structural and biological functions performed by canonical linker histones in later development, may be shared in early embryos by weakly homologous proteins, such as BigH1, or even unrelated, non-homologous proteins, such as Elba2.

DNA in the nuclei of all eukaryotic cells is packaged into a compact nucleoprotein complex called chromatin^1^,^2^. Chromatin is organized into repeating units of nucleosomes that constitute the fundamental structural unit of chromatin. Each nucleosome consists of an octamer of two molecules of each of the four core histones H2A, H3B, H3 and H4 around which is wrapped ~145 bp of DNA. Chromatin also contains a fifth type of histone usually referred to as the linker histone H1. H1 binds to nucleosomes as well as the DNA between nucleosomes (linker DNA) and protects an additional ~20 bp of the linker DNA. *In vitro* studies indicate that binding of H1 to oligonucleosomal arrays stabilizes the association of DNA with the core histone octamer and facilitates the folding of the arrays into more compact structures^3^,^4^. Binding of H1 also increases the spacing between nucleosomes and restricts their mobility. Thus, *in vitro* studies indicate that H1 plays key roles in the structure of the chromatin fiber. This view is supported by a limited number of studies *in vivo*. For example, studies of *Drosophila* larvae that have been depleted of H1 by RNAi show marked changes in polytene chromosome structure including misalignment of sister chromatids, as well as changes in the structural integrity of heterochromatin, including the deposition of its characteristic histone marks (H3K9me2 and H4K20me2)^5^. Additional support for H1 as a key structural component of chromatin comes from the fact that the stoichiometry of H1 in chromatin ranges from 0.5 to nearly 1 in a wide variety organisms and cell types^6^. However, recent evidence indicates that H1 functions in chromatin involve more than its structural contributions. H1 interacts with a large number of chromatin-associated proteins^7^. Although in most instances the functional significance of such interactions have not yet been defined, in a few cases interactions of H1 with other proteins have been shown to be required for H1-mediated processes in chromatin. For example, H1 interacts directly with the H3K9-specific histone methyltransferase Su(var)3–9 and recruits it to chromatin to promote H3K9 methylation of pericentric heterochromatin and repression of transposable element transcription^8^,^9^.

Although histones are highly conserved proteins, most multicellular organisms express several variants of each type of histone, except H4. Among the histone classes, the H1 linker histones are the most divergent group. For example, mammals express 11 H1 variants^10^, some of which appear to have overlapping or redundant functions^11^. Some of the mammalian H1 variants exhibit tissue-restricted expression. For example, the murine oocyte-specific linker histone (H1oo) is present exclusively in oocytes and very early embryos^12^. Considering that many organisms express multiple H1 variants, *D. melanogaster* is quite unique, since it expresses only a single H1 protein during most of its development. Recently, an H1-like protein called dBigH1 was identified in *Drosophila*^13^. BigH1 is expressed prior to the cellular blastoderm stage of embryogenesis, a period when *Drosophila* H1 is not detectable and appears to confer on chromatin some of the structural features associated with H1. However, whether it is able to substitute for H1 has not been tested. The discovery of BigH1 also raises the question whether other proteins with H1-like properties remain to be discovered in *Drosophila*.

The BEN domain (BEND) is an ~90-amino-acid α-helical module conserved in diverse polydnavirus and cellular metazoan proteins, such as human BANP/SMAR1, NAC1 and *Drosophila* Mod(mdg4)^14^. A family of “BEN-solo” factors is characterized by the presence of BEND as a single conserved module of the proteins^15^. However, BEN domains frequently appear in tandem copies of two to four or are linked to other evolutionary conserved motifs (BTB/POZ, coiled-coiled regions, C4DM, C2H2 fingers, etc). Based on contextual conservation of BEND-containing proteins, it was predicted that they function as DNA-binding factors or adaptor molecules that recruit chromatin-modifying complexes^14^.

*Drosophila* BEN solo protein Insv promotes peripheral nervous system development, acts as a nuclear corepressor for Su(H), a Notch transcription factor^16^, and is recruited to chromatin via binding to the CSL-type transcription factor, a primary effector of Notch signaling^17^. Mammalian proteins BEND5 and BEND6 are highly homologous to Insv. Murine BEND5 is strongly expressed in the brain cortex, and human BEND5 can substitute for Insv in transient transfection assays^15^. On the other hand, BEND6 exhibits key attributes of a true functional ortholog of Insv: it binds CSL, associates with and represses Notch targets and restricts Notch signaling in neural stem cells^18^. Consistent with these properties, a BEND6 transgene is able to rescue the *insv* phenotypes *in vivo*^19^. Genome-wide (ChIP-seq) analyses of Insv distribution and reporter assays suggested that it binds with a high specificity to a novel recognition motif (TCYAATHRGAA)^15^. The crystal structure of an Insv BEND-DNA target complex revealed homodimeric association of the BEN domain and specific DNA binding through extensive nucleotide contacts with its α helices and C-terminal loop^15^. Thus, BEND was postulated to function in a DNA sequence-specific manner.

Recently, we performed a mis-expression genetic screen for *Drosophila* modifiers of *His1*^20^. One of the strongest suppressors of lethality due to H1 depletion was a *UAS* allele of *Elba2*, which encodes a BEN solo protein. Elba2 was originally characterized in studies that described sequence-specific DNA affinity purification of *Drosophila* embryonic proteins that bind to the *Fab-7* chromatin boundary element of the Bithorax complex^21^. Two BEN solo proteins, Elba1/Bsg25A and Elba2, together with another polypeptide Elba3 exhibited high-affinity, cooperative binding to the *Fab-7* element *in vitro* and were proposed to form a tripartite complex (Elba) that functions as an insulator factor in early embryogenesis^21^. Among other outcomes of our genetic screen, we expected to identify factors that could, when ectopically overexpressed, replace multiple functions of H1 and thus relieve the stringent requirement for its high-level constitutive expression. Elba2 is a small (43 kDa) basic (pI ~9.8) protein. It is an abundant chromatin component in *Drosophila* embryos but it is not strongly expressed in larvae^21^. Therefore, we decided to check whether ectopic ubiquitous expression of Elba2 could rescue cellular/chromosome defect phenotypes associated with H1 depletion in L3^5^. In this paper, we demonstrate that ectopic overexpression of Elba2 in larvae strongly complements all known phenotypes of “hypomorphic” *His1* RNAi alleles, and thus, Elba2 can partially substitute for multiple functions of H1 *in vivo*.

## Results

### *Elba2* expression is up-regulated in the UAS/GAL4-dependent mis-expression allele *P{EP}Elba2* ^*G17999*^

**Figure 1:**
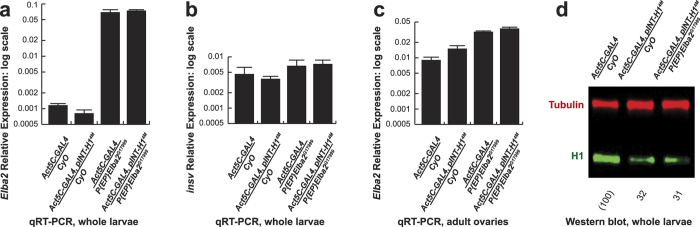
Regulation of *Elba2*, *insv* and *His1* by the *UAS-Elba2* mis-expression allele. **(a)** *Elba2* is strongly up-regulated in the *Act5C-GAL4/P{EP}Elba2*^*G17999*^ background. *Elba2* mRNA expression level (relative to that of *rp49*) in whole L3 larvae of various genotypes was measured by quantitative RT-PCR. Error bars, standard deviation. (**b**) *insv* expression is not substantially affected in the *Act5C-GAL4/P{EP}Elba2*^*G17999*^ background. *insv* mRNA expression was analyzed as in **(a)**. Error bars, standard deviation. (**c**) *Elba2* expression is only moderately increased in *Act5C-GAL4/P{EP} Elba2*^*G17999*^ ovaries. *Elba2* mRNA expression in was measured in adult ovaries as in **(a)**. Error bars, standard deviation. (**d**) Depletion of H1 protein by RNAi is not rescued in the *Act5C-GAL4/P{EP}Elba2*^*G17999*^ background. H1 protein (green) expression was measured by quantitative western blot of lysates prepared from whole larvae of the indicated genotypes. Tubulin (red) served as loading control.

Abrogation of H1 expression by RNAi *in vivo* leads to a significant reduction of adult fly eclosion rates due to lethality at the larval-pupal transition^5^. Depending on the strength of the RNAi allele and temperature, adult viability is affected over a wide range, from a moderate decrease to complete lethality. We have recently identified a number of *Drosophila His1* modifiers in a lethality-based genetic screen of *EP* misexpression alleles^20^. Among them, *P{EP}Elba2*^*G17999*^ strongly suppresses lethality caused by moderate depletion of H1 by RNAi (to ~50% of wild-type levels). The allele harbors a *P{EP}* insertion^22^ on the second chromosome in the intergenic region between *Insv* and *Elba2* genes. Suppression of H1 depletion phenotypes by *P{EP}Elba2*^*G17999*^ could be caused by up-regulation of Insv and/or Elba2. To assign the suppressor function to a specific gene, we analyzed the expression of *insv* and *Elba2* transcripts in whole L3 larvae by quantitative RT-PCR (Fig. 1a,b). The combination of the ubiquitous *Act5C-GAL4* driver with *P{EP}Elba2*^*G17999*^ results in strong (>50-fold) activation of *Elba2*, relative to wild-type controls. In contrast, *insv* expression is not appreciably increased (<2-fold). Thus, *P{EP}Elba2*^*G17999*^ is a specific mis-expression allele of *Elba2*. Additionally, H1 depletion by RNAi does not substantially affect the expression of *Elba2* in control or mis-expression backgrounds.

According to MODENCODE RNA-seq data, endogenous *Elba2* is only weakly expressed in whole L3 larvae. On the other hand, it is strongly expressed in adult ovaries and early (0–4 hr) embryos, where it is presumably maternally loaded. We decided to compare the *Elba2* expression level in *Act5C-GAL4/ P{EP}Elba2*^*G17999*^ larvae to the physiologically relevant level in wild-type ovaries (Fig. 1c). Interestingly, the maximal ovarian *Act5C-GAL4*-driven mRNA expression in the *UAS* allele was close to that in whole larvae and only 2–3 times higher than wild-type control levels. Therefore, ectopic overexpression of *Elba2* under the control of the ubiquitous *Act5C-GAL4* driver in whole larvae, although much higher than in wild type, is comparable to normal levels of endogenous *Elba2* expression in ovaries.

### *Elba2* overexpression does not reverse depletion of H1 protein by RNAi

One explanation for suppression of H1 depletion-related phenotypes by *P{EP}Elba2*^*G17999*^ is an effect on H1 expression. Since Elba2 has been hypothesized to act as a DNA-binding transcription and/or insulator factor^21^, it is possible that its overexpression results in activation of *His1* transcription, causing a reversal of RNAi-dependent abrogation of *His1* expression. To test this hypothesis, we examined H1 protein levels in H1 RNAi-depleted larvae with and without *Elba2* overexpression (Fig. 1d). RNAi decreases H1 expression >3-fold, relative to wild-type control, however *P{EP}Elba2*^*G17999*^ had no detectable effect on H1 expression. We conclude that *Elba2* function is non-epistatic with that of *His1*, and the genetic interaction between them is due to similar but independent effects of their encoded proteins on downstream cell function(s) *in vivo*.

### Reduced viability of H1-depleted flies is rescued by ectopic expression of Elba2

To examine the effect of Elba2 overexpression on adult viability, we crossed recombinant *Act5C-GAL4, pINT-H1*^*4M*^*/SM5* females to homozygous *P{EP}Elba2*^*G17999*^ males at 27 °C and examined the relative numbers of *Cy*^+^ (H1-depleted) and *Cy* (H1-normal) adults in the offspring (Table 1). In these conditions (and in all other experiments), H1 was depleted to 30–50% of wild-type levels^5^,^8^. *w*^*1118*^ males or *Act5C-GAL4/SM5* females were substituted in control crosses. Indeed, we observed strong and statistically significant Elba2-dependent suppression of lethality caused by H1 depletion. The complementation effect can be recapitulated by Elba2 overexpression produced by an independent *UAS-Elba2* transgenic insertion introduced on the second chromosome^19^.

Next, we investigated whether suppression of the H1 RNAi-induced lethality by *Elba2* is specific. We observed that similar UAS-driven overexpression transgenes for Insv or its mammalian functional counterpart BEND6^19^ did not lead to suppression of lethality (Table 1). In fact, ectopic overexpression of Insv strongly enhanced the adult lethality. Thus, the functional interaction between H1 and Elba2 is specific and not shared by other BEND proteins. Accentuation of the H1 RNAi-induced lethality by the *UAS-insv* transgene may arise in part from a “dominant negative” effect of Insv overexpression, which may lead to interference with endogenous Elba2 function in attenuating the H1 depletion phenotypes.

### *Elba2* is not essential but exhibits genetic interactions with His1

**Figure 2:**
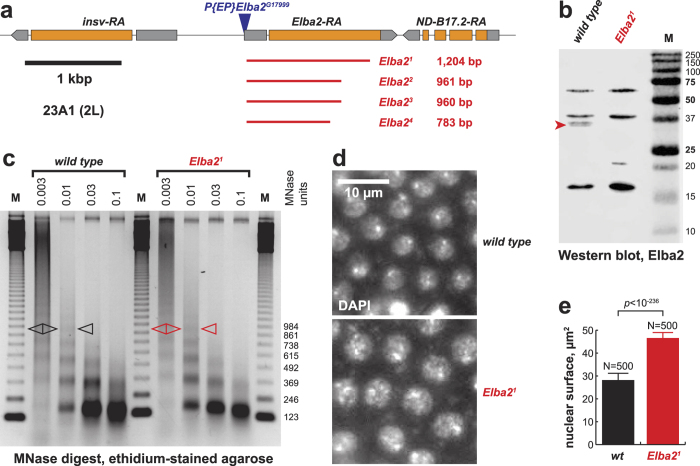
The function(s) of Elba2 in global compaction of embryonic chromatin. (**a)** Schematic of the *Drosophila Elba2* locus. A 6-kbp genomic interval in *D. melanogaster* 23A1 cytological region (left arm of chromosome 2) encompassing *insv*, *Elba2* and *ND-B17.2* genes is shown. Blue triangle, *P*-element insertion; red lines, genomic deficiencies in corresponding mutant *Elba2* alleles; thick black line, scale bar, 1 kbp; thin black lines, introns and intergenic regions; gray boxes, non-coding parts of exons; orange boxes, coding regions of exons; exon-intron boundaries are depicted as identified for major gene transcripts (named in the top line). (**b**) *Elba2*^*1*^ is a null mutant allele. Lysates of nuclei from 0–4 h embryos (homozygous *Elba2*^*1*^ and isogenic wild-type control) were analyzed by immunoblotting. A polypeptide with an apparent molecular mass of ~36 kDa (red arrowhead) is recognized by mouse anti-Elba2 antibody in wild-type but is absent in *Elba2*^*1*^ embryos. M, molecular mass marker; marker sizes (kDa) are shown on the right. (**c)** Nucleosome repeat length (NRL) is slightly reduced in chromatin of homozygous *Elba2* null mutant embryos. Samples of nuclei from wild-type control and *Elba2*^*1*^ embryos were subjected to partial digestion with the indicated number of units of micrococcal nuclease (MNase), and the DNA was analyzed by agarose gel electrophoresis and EtBr staining. Pentanucleosome bands are calculated to be 947 bp long in wild type (black open triangles) and 910 bp long in *Elba2*^*1*^ (red open triangles). M, 123-bp DNA ladder; marker sizes are shown on the right. (**d**) *Elba2* mutation leads to a decrease of nuclear sizes in syncytial embryos. Wild-type control and homozygous *Elba2*^*1*^ null embryos were stained with DAPI. DAPI-stained nuclei of an embryo in the interphase of nuclear division cycle 12 are shown. Scale bar (white), 10 μm. (**e**) The sizes of interphase nuclei in *Elba2* null syncytial embryos are dramatically decreased. Surface areas of interphase nuclei (division cycles 11–13) were measured in homozygous *Elba2*^*1*^ (red bar) and isogenic wild-type control (black bar) embryos based on DAPI staining (**d**). Mean values (μm^2^) are plotted. Error bars, standard deviations; N = 500 (10 nuclei each in 50 randomly selected embryos); probability value was calculated by two-tailed paired Student T-test.

To analyze the biological functions of Elba2 *in vivo*, we generated mutant alleles of *Elba2* by imprecise excision of *P{EP}Elba2*^*G17999*^. We recovered four deficiency alleles that contain deletions of 783 to 1,204 bp downstream of the *P*-element insertion site (Fig. 2a). In the largest deficiency, *Df(2L)Elba2*^*1*^, 1,204 bp (−108 to +1,086 relative to the start codon of *Elba2*) are excised and replaced with a 26-bp fragment of the *P*-element. Thus, almost the entire coding sequence, with the exception of the last C-terminal 19 codons, is eliminated. Therefore, *Elba2*^*1*^ is a null allele.

First, we checked whether *Elba2* mutations alone affect fly viability. To this end, we crossed heterozygous *Elba2/CyO* flies *inter se* and examined the distribution of *Cy*^+^ and *Cy* genotypes in the F1 progeny (Table S1). It appears that *Elba2* is not essential. Significantly, there was no appreciable effect of *Elba2* mutations on fly development or adult eclosion, since the relative proportion of *Elba2/Elba2* homozygotes in the offspring of the crosses was very close to the expected Mendelian ratio. Also, the homozygous mutant flies (both males and females) produce normal progeny in crosses with wild-type counterparts or *inter se* and are thus, fertile. Since homozygous *Elba2*^*1*^ animals are viable and fertile, we sought to confirm that they do not express Elba2 protein. To this end, we raised polyclonal antibodies to full-length recombinant Elba2 and used them for immunochemical analyzes of 0–4 h embryos, where endogenous Elba2 RNA is highly expressed^21^. We observed a strong expression of a 36 kDa protein specifically recognized by Elba2 antibody in nuclei prepared from wild-type control embryos. However, its expression was not detected in *Elba2*^*1*^ embryos (Fig. 2b). Thus, as expected, *Elba2*^*1*^ embryos do not express Elba2.

The genetic interaction between *Elba2* and *His1* described above (Table 1) relies on artificial ectopic overexpression of *Elba2* in larvae, where the endogenous gene has limited transcriptional activity (Fig. 1a). Therefore, we decided to test whether endogenous *Elba2* and *His1* also exhibit genetic interactions. To this end, we examined how *Elba2* mutation affects viability of H1-depleted flies. We crossed *Tub-GAL4/TM3,Sb* and *pINT-H1*^*1M*^*/TM3, Sb* flies in homozygous *Elba2*^*1*^ and wild-type (isogenic, precise excision) backgrounds and scored the distributions of H1-depleted (*Sb*^+^) and control (*Sb*) eclosed adults in the progeny. We discovered that the *Elba2* null mutation significantly reduced viability of H1-depleted flies (Table 2). Thus, as expected, *Elba2* null and hypomorphic *His1* mutations exhibit a strong synthetic lethal interaction. Abrogation of H1 expression results in more severe lethality when performed in the *Elba2* null background. Therefore, endogenous Elba2 partially compensates for H1 depletion *in vivo*.

### Elba2 contributes to genome-wide chromatin compaction

Although Elba2 is not essential for viability, we investigated its effects on chromatin structure in early embryos, where it is strongly expressed^21^ (and where the loading of canonical H1 into chromatin is low^13^,^23^). We collected nuclei from 0–4 h *Elba2*^*1*^ and *Elba2*^+^ embryos and examined their nucleosome structure by partial micrococcal nuclease (MNase) digestion assay (Fig. 2c). We observed a small but reproducible decrease of nucleosome repeat length (NRL) in embryos that do not express Elba2 (from ~189 bp in wild type to ~182 bp), comparable to that observed in animals with reduced expression of prototypical linker histones H1 and BigH1^5^,^13^. Therefore, similar to linker histone H1, Elba2 is required to maintain a normal NRL of bulk native chromatin and, similar to BigH1, controls global chromatin structure in early embryos.

We also noticed that nuclear pellets from *Elba2*^*1*^ and *Elba2*^+^ embryos containing equivalent amounts of nucleic acid differed in their volumes by nearly two-fold. Nuclei prepared from *Elba2* mutants appeared consistently less dense than wild-type nuclei. To determine whether mutant *Elba2* embryos have larger nuclei *in vivo*, we stained *Elba2*^*1*^ and *Elba2*^+^ embryos (0–4 h AED) with DAPI (Fig. 2d) and measured the diameters of interphase syncytial nuclei (division cycles 11–13) based on DAPI staining (Fig. 2e). Whereas, the sizes of nuclei were relatively uniform within each genotype, nuclei in *Elba2*^*1*^ embryos were on average ~29% larger than nuclei in wild-type embryos (linear size, equivalent to an ~65% increase of the measured surface). We conclude that Elba2 plays an important role in global compaction of chromatin in nuclei of *Drosophila* embryos.

### Elba2 distribution in chromatin is nearly ubiquitous and overlaps with that of H1

**Figure 3:**
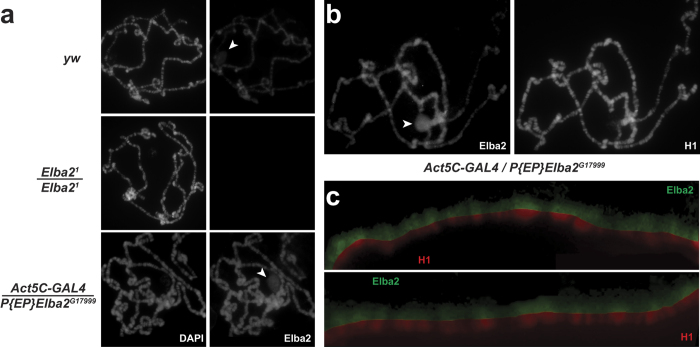
Elba2 distribution in polytene chromosomes. (**a**) Endogenous and ectopically expressed Elba2 is nearly ubiquitously distributed in polytene chromosome arms. Polytene chromosomes of salivary glands from control (*yw*), *Elba2* null and *Elba2*-ectopically expressing larvae were stained with DAPI and affinity-purified Guinea pig anti-Elba2 antibodies. No Elba2 signal above background is detected in the *Elba2* null. Arrowheads, nucleoli. (**b**) Elba2 distribution in polytene chromosomes is restricted to polytene bands and resembles that of H1. Elba2 and H1 distribution in polytene chromosomes from *Elba2*-ectopically expressing larval salivary glands were examined by IF as in **(a)**. Arrowhead, nucleolus. (**c**) Elba2 and H1 exhibit strong co-localization in polytene chromosomes. Split-images of fragments of polytene chromosomes analyzed by indirect immunofluorescence with Elba2 (green) and H1 (red) antibodies.

Elba2 and other BEND proteins were proposed to function as sequence-specific, DNA-binding factors based on structural considerations^15^. Also, Elba2 was originally purified as a component of a putative sequence-specific insulator factor Elba^21^. These findings suggest that Elba2 may exhibit a restricted distribution pattern in the genome, limited to loci that contain its recognition sequence(s). To examine Elba2 distribution in chromatin *in vivo*, we used Elba2 antibodies to analyze the distribution of the protein in larval polytene chromosomes by indirect immunofluorescence (IF) microscopy. Endogenous Elba2 can be readily detected by IF staining in wild-type salivary gland cells (Fig. 3a, top). Unexpectedly and contrary to the predicted restricted distribution of Elba2, the observed pattern of Elba2 occupancy is nearly ubiquitous, and the protein localizes mostly to the bands of polytene chromosomes. In contrast, a prototypical insulator protein Su(Hw)^24^ exhibits a substantially more discrete distribution pattern, consistent with its known locus-specific functions (Fig. S1). Thus, Elba2 does not associate with chromatin in the locus-specific manner expected of a transcription and/or insulator factor. Alternatively, its binding site recognition specificity may be very relaxed.

The observed IF staining of Elba2 is specific, since no signal can be detected in homozygous *Elba2*^*1*^ null mutant (Fig. 3a, middle). The intensity of the staining is also moderately increased in polytene chromosomes from L3 larvae that ectopically overexpress Elba2 (in *Act5C-GAL4/P{EP}Elba2*^*G17999*^ animals, Fig. 3a, bottom). Importantly, *Elba2* mutation or overexpression does not affect the overall morphology of polytene chromosomes (compare DAPI images in left panels in different genetic backgrounds).

Since endogenous and ectopically expressed Elba2 localizes predominantly to polytene chromosome bands, which contain condensed, repressed chromatin, it is possible that Elba2 loading into chromosomes overlaps with that of H1. Thus, we performed co-staining of polytene chromosomes with Elba2 and H1 antibodies (Fig. 3b). We observed a substantial correlation of Elba2 and H1 IF signals. In high-resolution split images (Fig. 3c), it is clear that Elba2 and H1 patterns are very similar: the majority of H1-negative loci show greatly reduced levels of Elba2 staining and *vice versa*. In contrast, high-abundance loci for both proteins strongly correlate. The co-localization of Elba2 and H1 in polytene chromosomes suggests a model of direct replacement of H1 by Elba2 upon simultaneous H1 depletion and Elba2 overexpression. When Elba2 is ectopically expressed in H1-depleted animals, it is deposited in loci formerly occupied by H1 and provides repressive and/or chromatin effector recruitment function(s) to partially compensate for the loss of H1.

### Ectopic expression of Elba2 ameliorates defects of chromosome structure that are associated with H1 depletion

Depletion of H1 in salivary gland cells results in profound defects of polytene chromosome architecture: the regular band-interband pattern of aligned chromatin fibers is compromised, and the normal singular heterochromatic chromocenter is dissociated into multiple diffuse foci containing HP1^5^. Furthermore, abrogation of H1 expression almost completely eliminates IF staining of polytene chromosomes with H3K9me2-specific antibodies. The latter effect is attributed to the reduced/abolished tethering of the H3K9 methyltransferase Su(var)3–9^8^. Together, these molecular defects likely contribute to the larval/pupal lethality of H1-depleted animals^9^.

**Figure 4:**
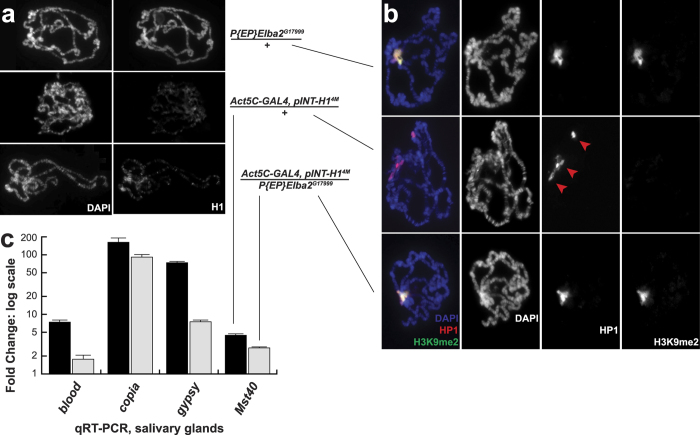
Rescue of H1 depletion phenotypes *in vivo* by ectopic expression of Elba2. (**a)** *Elba2* mis-expression restores regular polytene band-interband structure in salivary glands depleted of H1 by RNAi. Polytene chromosomes of salivary glands from control, H1-depleted larvae and larvae that are simultaneously depleted of H1 and ectopically expressing *Elba2* were stained with DAPI and H1 antibodies. (**b**) Polytene chromosome defects due to H1 depletion *in vivo* are ameliorated by ectopic mis-expression of *Elba2*. Polytene chromosomes prepared from animals of the indicated genotypes as in **(a)** were stained with DAPI (blue) and antibodies against HP1 (red) and H3K9me2 (green). Red arrowheads, multiple HP1-rich foci that do not coalesce into a singular chromocenter in H1-depleted salivary glands. (**c)** Derepression of transposable elements upon H1 depletion is partially reversed by ectopic expression of *Elba2*. Transcripts of the indicated TE’s in salivary glands were measured by quantitative RT-PCR in control (*P{EP}Elba2*^*G17999*^*/*+), *Act5C-GAL4, pINT-H1*^*4M*^*/*+ and *Act5C-GAL4, pINT-H1*^*4M*^*/P{EP}Elba2*^*G17999*^ backgrounds. Transcript levels were normalized to that of *rp49*, and the fold change brought about by H1 depletion (black bars) and H1 depletion with simultaneous *Elba2* overexpression (gray bars) was calculated relative to the control allele. All RT-PCR reactions were performed on three independent biological samples in duplicate. Significance testing was performed by applying two-tailed paired Student T-test to normalized ∆Ct values (with and without ectopically expressed Elba2); *p*-values are <0.02 for all transcripts. Error bars, standard deviation.

We investigated whether these abnormalities are rescued by ectopic expression of Elba2. First, we compared the gross morphology of polytene chromosomes in wild-type control and H1-depleted salivary gland cells, as well as in cells where H1 depletion was accompanied by Elba2 overexpression. To this end, we performed DAPI staining of polytene spreads from various progeny of crosses between *Act5C-GAL4, pINT-H1*^*4M*^*/T(2;3)B3, CyO: TM6B, Tb* and *P{EP}Elba2*^*G17999*^*/P{EP}Elba2*^*G17999*^ or *w*^*1118*^ parents (Fig. 4a). In addition, to verify H1 depletion, we stained these preparations with an H1-specific antibody. Whereas control polytene spreads (exemplified by *P{EP}Elba2*^*G17999*^*/T(2;3)B3, CyO: TM6B* or *w*^*1118*^ animals) exhibited regular banded structure (Fig. 4a, top), the banding pattern was severely disrupted in *Act5C-GAL4, pINT-H1*^*4M*^*/*+ salivary glands (Fig. 4a, middle). However, the band-interband structure was restored to normal when the H1 depletion was combined with Elba2 overexpression in *Act5C-GAL4, pINT-H1*^*4M*^*/ P{EP}Elba2*^*G17999*^ animals (Fig. 4a, bottom). Similarly, the singular chromocenter that is easily identified in DAPI-stained control polytene spreads is not apparent in H1-depleted polytene chromosomes but restored upon Elba2 overexpression. Consistent with immunoblot analyses (Fig. 1d), H1 expression or deposition into chromatin is not substantially elevated in *P{EP}Elba2*^*G17999*^-rescued chromosomes in comparison to H1-depeletd chromosomes (Fig. 4a, right panels).

We also examined these polytene spreads for deposition of the heterochromatin-specific histone mark H3K9me2 and distribution of HP1 (Fig. 4b). As described previously^5^, H1 depletion disrupts the singular chromocenter and produces multiple HP1-rich foci in polytene spreads; it also severely reduces polytene chromosome staining with anti-H3K9me2 antibodies (compare top and middle panels of Fig. 3b). In contrast, simultaneous depletion of H1 and ectopic expression of Elba2 reverses the two phenotypes to that of the control (Fig. 4b, bottom). Thus, ectopically overexpressed Elba2 partially compensates for the loss of H1 and ameliorates all of the described microscopic defects of polytene chromosome structure that are caused by H1 depletion in larvae.

### H1 RNAi-dependent derepression of transposable elements is reversed upon ectopic expression of Elba2

Normal levels of H1 in chromatin are essential for the proper regulation of genetic activity in flies. Reducing the abundance of H1 leads to positive and negative effects on expression of multiple *Drosophila* genes in salivary glands and Kc cells^8^. In particular, H1 depletion causes an extremely strong (up to 600-fold) derepression of transposable elements (TE’s) and other repetitive sequences. This up-regulation is partially reversed by overexpression of Su(var)3–9^8^. Since H3K9 methylation is restored in H1-depleted animals also expressing ectopic Elba2 (Fig. 4b), we determined whether Elba2 overexpression in salivary glands also reverses derepression of TE’s in H1 RNAi animals. We observed a significant (3- to 20-fold) negative effect of Elba2 ectopic expression on TE transcript levels in H1-depleted salivary glands (Fig. 4c). Thus, ectopic Elba2, like H1 itself, promotes repression of repetitive sequences and counteracts the derepression of these elements occurring upon H1 depletion.

### Ectopic BigH1 or Elba2 have similar restorative activity in reversing lethality and defects in chromosome structure in H1-depleted larvae

We showed previously that ectopic expression of *His1* cDNA transgenes and duplications of the histone gene cluster can partially rescue lethality due to the RNAi-mediated abrogation of H1 expression^5^,^9^. The preceding series of experiments demonstrate that ectopic expression of Elba2 also compensates for RNAi-mediated loss of endogenous H1, resulting in rescue of lethality and chromosomal defects (Table 1 and Fig. 4). Therefore, ectopic Elba2 can assume several of the biological functions of H1. Recently, an H1-like protein dBigH1 was identified in *Drosophila*^13^. Its expression, similar to that of Elba2, is largely limited to ovaries and early embryos. We sought to determine whether ectopic ubiquitous expression of BigH1 in larvae counteracts the effects of H1 depletion. To this end, we used the existing *P{EP}BigH1*^*G18579*^ *UAS* misexpression allele in which *BigH1* transcription can be stimulated by GAL4. We observed that ectopic expression of BigH1 under the control of Act5C-GAL4 driver rescues viability of H1-depleted adults (larvae) (Table 1). Therefore, the *P{EP}Elba2*^*G17999*^ and *P{EP}BigH1*^*G18579*^ alleles phenocopy each other, and mis-expression of either Elba2 or BigH1 complements a deficiency of H1 protein.

We then examined the ability of ectopically expressed BigH1 to rescue chromosome defects in H1-depleted salivary glands. We stained *Act5C-GAL4, pINT-H1*^*4M*^*/*+*; P{EP}BigH1*^*G18579*^*/*+ polytene chromosomes with DAPI, HP1 and H3K9me2 antibodies and observed a normal chromosome architecture, including regular band-interband structure, single chromocenter and strong dimethylation of H3K9, identical to that seen in wild type polytene chromosomes (Fig. S2). Therefore, Elba2 and BigH1 have very similar biological activities such that ectopic expression of either protein can substitute for H1 when it is depleted by RNAi *in vivo*.

## Discussion

Although BEN domain proteins were proposed to function as sequence-specific factors^15^, the relationship between their DNA binding activity and distribution in the genome *in vivo* remains enigmatic. For instance, reporter assays indicate that *Drosophila* Bsg25A and Elba2 polypeptides can individually recognize the palindromic Insv DNA binding motif with high affinity. Furthermore, the crystal structure of the Bsg25A BEND-DNA complex suggests that Bsg25A shares key aspects of DNA binding *in vitro* with Insv^19^. On the other hand, the *Fab-7* Elba motif, the presumed *in vivo* target of Bsg25A and Elba2, is quite distinct from the Insv site^19^,^21^. Also, Insv exhibits extensive co-binding with class I insulator elements that generally do not conform to its binding consensus^19^. Of interest, mammalian BEND5-VP16 fusion protein can specifically activate 4x-Insv reporters in transient transfection assays, and GST-BEND5 can bind EMSA probes that bear an intact Insv motif^15^. On the other hand, similar fusions of mammalian BEND6 fail to perform in these assays^19^, yet a BEND6 transgene strongly complements homozygous *insv* mutation in flies *in vivo*^19^. Therefore, BEND proteins may utilize alternative mechanisms for tethering to their functional sites in the genome, such as recruitment by adapter proteins or deposition by specialized chaperones.

In this work, we show that *Drosophila* BEND protein Elba2 exhibits a distribution pattern in salivary gland polytene chromosomes that is inconsistent with its putative sequence-specific binding. Unambiguously, instead of restricted binding to a number of discrete bands that would be expected of a sequence-specific factor, Elba2 is broadly distributed to virtually all polytene bands, which mostly represent compact, silent chromatin also occupied by the linker histone H1 (Fig. 3). It is possible that sequence recognition by Elba2 is very relaxed, and thus, it can tolerate substantial degeneracy among its binding sites. Alternatively, deposition of Elba2 in chromosomes may rely on completely sequence-independent mechanisms.

The postulated global, genome-wide function of Elba2 *in vivo* is further supported by the effect of the endogenous protein on the chromatin NRL and nuclear compaction in early embryos (Fig. 2). Although the overall oligonucleosome structure and periodicity is not affected in the bulk native chromatin of *Elba2* mutant embryos, the nucleosomes are more closely spaced, like those in H1-depleted larvae^5^ and *BigH1* mutant embryos^13^. The decrease of NRL in *BigH1* mutant embryos was reported to be more substantial than that in *Elba2* embryos (17 bp versus 7 bp) and even that in larvae depleted of about 95% of H1^5^. However, differences in the methods used for quantifying NRL changes in the study of *BigH1* and the other studies (including the current work) may account for the discrepancy. Interestingly, we also observe that *Elba2* null mutation leads to a significant increase of the volume of syncytial nuclei, which further supports a global role for Elba2 in chromatin condensation. Although at the present time, we cannot exclude an indirect effect of Elba2 on the compaction of nuclear DNA, other evidence indicates that Elba2 functions via mechanisms similar to those of linker histone H1.

The expression of endogenous Elba2 protein is limited to the early embryo^21^; it is only weakly expressed in larvae (e.g., Fig. 1). On the other hand, H1 is expressed ubiquitously throughout development, and abrogation of its expression in L3, results in a dramatically reduced rate of adult eclosion. Upon moderate to strong depletion of H1, lethality occurs during the larval to pupal transition. We demonstrate here that the Elba2 polypeptide, when ectopically expressed in larvae under the control of a ubiquitous driver, can compensate for the loss of H1 *in vivo* (Table 1). Importantly, ubiquitous mis-expression of Elba2 rescues all known phenotypes observed in H1-depleted animals^5^,^8^, including polytene chromosome structure abnormalities as well as changes in the composition and genetic activity of heterochromatin (Fig. 4). Thus, Elba2 is expected to share multiple biochemical activities with H1: it may compact an oligonucleosome fiber *in vitro* and may also physically interact with natural H1 partner proteins, such as Su(var)3–9 and STAT92E^8^,^25^. Unfortunately, we were unable to express and purify recombinant Elba2 polypeptide in a soluble form. Both *E. coli* and *S. frugiperda* (baculovirus) expression systems produced highly insoluble Elba2 under several tested conditions (*not shown*), which hampered further biochemical experimentation. It is possible that native Elba2 in solution exists as a part of a heteromeric complex and thus, critically depends on the presence of additional subunits for solubility. In fact, Elba2 has been proposed to form a heterotrimeric complex Elba^21^. It has been demonstrated that Elba subunits synergistically bind to the *Fab-7* insulator element *in vitro*. However, (i) the existence of a stable Elba complex was not further confirmed, (ii) its complete composition is unknown, and (iii) Elba (or an Elba-like complex) has not been demonstrated to be the major complex of Elba2. In the future, it will be interesting to characterize the major native form of Elba2, using an unbiased approach that does not rely on the use of a sequence-specific DNA affinity resin. A complex of Elba2 (Elba or an alternative complex) may modify the structure of the chromatin fiber *in vitro* and/or physically interact with H1 partner proteins. In addition, it remains to be tested whether the nearly ubiquitous deposition of Elba2 in chromatin (Fig. 3) takes place in the context of a putative complex or as an individual polypeptide.

Most multi-cellular eukaryotes, including animals and plants, express several variants of linker histone H1, as many as 11 in mammals^10^. Although their variant-specific biological/biochemical functions are largely unknown, H1 variant genes exhibit heterogeneity in tissue-, cell cycle- and developmental stage-specific expression patterns. For example, murine H1oo protein is expressed only in the oocyte and very early embryo^10^,^12^. In contrast, Drosophilids typically express a single H1 isoform throughout their life cycle. The only known exception is *D. virilis*, which expresses three H1 variants. However, the differences amongst their sequences are more characteristic of polymorphic variants and therefore, it is likely that they are not true functional protein isoforms. Intriguingly, analysis of chromatin from very early *Drosophila* embryos (0–2 hours after egg deposition) indicates that H1 is not loaded into chromosomes at this stage^13^,^23^, when Elba2 is highly expressed. Thus, Elba2 may function as a “replacement linker histone” that carries out some of the normal biological activities of H1 in early embryonic chromatin, when canonical H1 is not present. Likewise, when ectopically expressed in larval cells with limiting H1 (depleted by RNAi), Elba2 is able to assume H1’s functions.

It has been proposed that a weakly homologous dBigH1 protein may function as an embryonic replacement H1 in *Drosophila*^13^. Importantly, the reversal of phenotypes associated with H1 depletion by ectopic overexpression of Elba2 is indistinguishable from that produced by ectopic overexpression of BigH1 (Table 1, Figs 4 and S2). Thus, at least in the context of reduced H1, ectopic Elba2 and BigH1 play nearly identical biological roles. The functional similarity *in vivo* between Elba2 and H1-homologous BigH1 further supports our idea that *Drosophila* Elba2 has features of an H1 replacement protein.

Surprisingly, both BigH1 and Elba2 are expressed in Drosophilids but lack true orthologs in other organisms. We surveyed several sequenced eukaryotic genomes and found a negative correlation between the conservation of BigH1 and Elba2 orthologs in a particular species and the existence of multiple linker histone H1 isoforms. Furthermore, BigH1 and Elba2 orthologs, when present in eukaryotic genomes, are always found together. It is possible that in ancient metazoan ancestor organisms, some of the functions of linker histone H1 may have been shared by proteins that are not structurally similar (Elba2) or only distantly related (BigH1) to H1. In early embryos, these proteins may perform biological functions (chromatin compaction and tethering of effector enzymes) that are characteristic of the canonical H1 during later stages of development. The emergence of H1 isoforms that are expressed early in development, such as H1oo in mammals, may have obviated the selective pressure to maintain H1 replacement proteins similar to *Drosophila* BigH1 and Elba2, which resulted in their evolutionary loss.

## Methods

### Fly strains and genetics

Flies were maintained on standard corn meal, sugar and yeast medium with Tegosept at 18–27 °C as indicated. *His1* RNAi and *P{EP}Elba2*^*G17999*^ alleles are described elsewhere^5^,^20^. *UAS-Elba2*, *UAS-insv* and *UAS-hBEND6* transgenes are a generous gift of Eric Lai (Memorial Sloan Kettering Cancer Center). *P{EP}BigH1*^*G18579*^, various balancer and GAL4 driver alleles were obtained from the Bloomington Stock Center. Genetic rescue experiments and adult fly viability analyses were performed at 27 °C as described previously^20^. See legends to Tables 1, 2 and S1 for details. To genotype L3 larvae that contain the *Act5C-GAL4, pINT-H1*^*4M*^ transgene combination, the allele was balanced with *T(2;3)B3, CyO: TM6B, Tb* translocation and used in crosses. *Tb*^+^ F1 progeny contain the recombinant second chromosome with the GAL4 driver and RNAi transgene.

*Elba2* null allele(s) were generated by imprecise excision of *P{EP}Elba2*^*G17999*^ insertion as described^26^. 107 *w*^*–*^ excision alleles were analyzed by genomic DNA PCR with the following primers: AGGTGGCATGAATCTGGGATAGCA and CAAGTACAAGTGGATAGCGGACCA. The size of wild-type PCR product is 3,198 bp. Four alleles with large deletions (>500 bp, see Fig. 2a) and several precise excision alleles (isogenic controls) were balanced with *CyO*; their genomic DNA flanking *Elba2* was amplified and sequenced. For viability tests, 10 heterozygous males and females each were crossed *inter se*, allowed to mate for 4 days and discarded; the distribution of balancer markers was scored in the adult progeny. For fertility tests, 10 homozygous males or females were crossed with 10 *yw* counterparts of the opposite sex, allowed to mate for 4 days and discarded. The appearance of advanced development stage/adult progeny of the crosses indicated fertility of *Elba2* males and females, accordingly.

To examine genetic interactions of *Elba2* and *His1*, synthetic alleles that harbor homozygous *Elba2*^*1*^ second chromosome and heterozygous balanced GAL4 driver or UAS-H1-RNAi transgenes on the third chromosome (*Elba2*^*1*^*; Tub-GAL4/TM3,Sb* and *Elba2*^*1*^*; pINT-H1*^*1M*^*/TM3, Sb*) were generated in a series of crosses. The two alleles were mated at 26 °C and the distribution of *Sb*^+^ (H1-depleted) and *Sb* (H1-normal) progeny was scored to assess the effect of H1 depletion in the *Elba2*^*1*^ background. For comparison, similar synthetic alleles with a precise excision allele (“+”) were generated and used in crosses.

### Real-time RT-PCR

Expression of transcripts for *Elba2*, *insv* and transposable elements was measured by quantitative RT-PCR on an ABI Prizm 770 sequence detection system (Applied Biosystems) as described previously^5^. Total RNA was prepared from 2–5 whole L3 larvae, 20 pairs of salivary glands or 20 adult ovaries dissected from animals of specific genotypes. The expression levels were quantitated relative to an endogenous reference gene, *rp49*. The following primers were used for *Elba2*: GATCAGGACTCGTGTCCTAACC and CGCTGGGCAGGATAGCAGTC; and *insv*: CGGACCCGCAAGTGGAGGTC and CATGAATCTGGGATAGCAGATCC, the primers for *rp49* and transposable elements *blood*, *copia*, *gypsy* and *Mst40* are described elsewhere^8^. All experiments were performed on three independent biological samples in duplicate, along with no-template controls.

### Partial micrococcal nuclease (MNase) digestion assay

Homozygous *Elba2*^*1*^ and *Elba2*^+^ embryos were collected 0–4 h after egg deposition (AED) and dechorionated. Nuclei were isolated and treated with MNase as described^5^. Briefly, ~200 mg of freshly prepared embryos were resuspended in 500 μl ice-cold Buffer A (15 mM Tris-HCl, pH 7.5, 15 mM NaCl, 60 mM KCl, 0.34 M sucrose, 0.5 mM spermidine, 0.15 mM spermine, 0.1% β-mercaptoethanol, 0.25 mM PMSF) additionally containing 2 mM EDTA and 0.5 mM EGTA and homogenized on ice using Roto-Dounce (Fisher Scientific). The nuclei were pelleted in a cold microcentrifuge (5 min at 13,000 rpm), washed twice with Buffer A (no EDTA/EGTA) and resuspended in 200 μl Buffer A. Nucleic acid concentrations were estimated by A_260_ measurement, and the volumes of two samples were adjusted with Buffer A to achieve equal concentrations. Aliquots of nuclei equivalent to 0.3 optical units (A_260_) were digested in four separate reactions with 0.003–0.1 units of MNase (Sigma) in a buffer containing 2 mM CaCl_2_ in a total volume of 50 μl for 10 min at room temperature. The digestions were stopped by adding EDTA to 25 mM, and nuclear RNA was degraded with 25 μg RNase A (Sigma) for 20 min at 37 °C. The samples were deproteinized by treating with Proteinase K and phenol-chloroform extraction as described^27^. DNA was precipitated with ethanol, and one-third of each sample was loaded on a 1.3% agarose gel in 1x TBE. The gel was stained with EtBr after electrophoresis.

### DAPI staining of *Drosophila* syncytial embryos

Homozygous *Elba2*^*1*^ and *Elba2*^+^ embryos were collected 0–4 h AED, dechorionated and fixed with methanol as described^28^. The embryos were mounted in Vectashield (Vector Laboratories) with 1 μg/ml DAPI and observed under a Zeiss Axiovert 200M. Fixed embryos were staged by the number of surface nuclei per unit area^29^. For each genotype, 50 embryos in the interphase of division cycles 11–13 were randomly selected, and a random area from the middle of each embryo was used to score the sizes of 10 blastoderm nuclei using AxioVision digital image processing software. Mean, standard deviation and two-tailed paired Student T-test values were calculated using Microsoft Excel.

### Antibodies, immunoblot analyses and immunohistochemistry

Full-length recombinant His-tagged Elba2 was expressed in *E. coli* and purified in denaturing conditions (8 M urea) on Ni-NTA agarose (Qiagen). Details of expression construct cloning and purification procedures are available upon request. After urea was removed by dialysis in PBS, the protein was precipitated out of solution, and the suspension of insoluble protein was used to raise polyclonal mouse (AECOM Hybridoma Facility) and Guinea pig antibodies (Pocono Rabbit Farm and Lab). Guinea pig immunoglobulins were purified from plasma using Bakerbond ABx (J. T. Baker). They were further affinity-purified on the resin obtained by cross-linking denatured Elba2 polypeptide to NHS-activated Sepharose (Pierce). Polyclonal rabbit anti-Su(Hw) antibody^24^ was a generous gift of V. Corces; other antibodies were previously described in ref. 5.

Western blotting was performed as described^5^ using whole larval L3 lysates or lysates of embryonic (0–4 h AED) nuclei in SDS-PAGE loading buffer. Polyclonal rabbit anti-H1 and mouse anti-Elba2 antisera as well as monoclonal mouse anti-tubulin E7 (loading control) and infrared dye secondary antibodies^5^ were used at 1:50,000, 1:1,000, 1:500 and 1:10,000 dilutions, respectively. Images were obtained and quantitated using a LI-COR Odyssey system.

Indirect immunofluorescence (IF) staining of polytene chromosomes was performed exactly as described^5^. Briefly, salivary glands of the wandering third instar larvae were dissected in PBS +0.1% Triton X-100. Polytene chromosomes were fixed in 3.7% paraformaldehyde for 30 sec, squashed in 45% acetic acid +3.7% formaldehyde, and frozen in liquid nitrogen. They were incubated overnight in PBS +10% fat-free milk +0.1%Triton X-100 with primary antibodies, washed twice in PBS +400 mM NaCl +0.2% NP-40 for 30 min and stained with secondary antibodies in PBS +0.1% Triton X-100. The final preparations were mounted in Vectashield mounting solution (Vector) and stained with DAPI (0.5 mg/mL). Affinity purified rabbit anti-H1, Guinea pig anti-Elba2 and rabbit anti-H3K9me2 (Abcam) were used at 1:5,000, 1:200 and 1:100 dilutions, respectively. Mouse anti-HP1 C1A9 antibody, rabbit anti-Su(Hw) and appropriate secondary goat Alexa Fluor (Molecular Probes) antibodies were used at 1:50, 1:200 and 1:200 dilutions, respectively. The preparations were examined using epifluorescence on a Zeiss Axiovert 200 microscope, and images were captured using a high-resolution CCD camera.

## Additional Information

**How to cite this article**: Xu, N. *et al.* BEN domain protein Elba2 can functionally substitute for linker histone H1 in *Drosophila* *in vivo.* *Sci. Rep.* **6**, 34354; doi: 10.1038/srep34354 (2016).

## Supplementary Material

Supplementary Information

## Figures and Tables

**Table 1:** Ectopic expression of Elba2 or BigH1 partially restores viability in H1-depleted animals.

Cross	*Act5C-GAL4/SM5*	*Act5C-GAL4, pINT-H1*^*4M*^*/SM5*	*p*-value
*w*^*1118*^ (*wt* control)	119/256 (128), 93%	24/187 (94), 26%	N/A
*P{EP}Elba2*^*G17999*^	125/246 (123), 102%	69/193 (97), 72%	**2.1** · **10**^**-7**^
*UAS-Elba2* (II)	153/295 (148), 104%	84/232 (116), 72%	**5.4** · **10**^**-8**^
*UAS-hBEND6* (II)	82/174 (87), 94%	33/195 (98), 34%	0.26
*UAS-insv* (II)	142/346 (173), 82%	2/234 (117), 2%	**3.9** · **10**^**-7**^
*P{EP}BigH1*^*G18579*^	93/219 (110), 102%	53/170 (102%), 102%	**2.6** · **10**^**-5**^

All crosses were performed at 27 °C. Males homozygous for UAS mis-expression EP alleles or UAS-driven transgenes on the II chromosome were mated to heterozygous *Act5C-GAL4/SM5, Cy* or *Act5C-GAL4, pINT-1-H1*^*4M*^*/SM5, Cy* females. Viability was scored as the number of eclosed *Cy*+ adults relative to the total number of offspring scored (columns 2 and 3). The expected numbers of *Cy*+ flies (calculated from the Mendelian distribution) are shown in parentheses; percent viability relative to the expected numbers is also shown. Low percentage numbers that indicate increased lethality are highlighted in red. Probability values are calculated by the chi-square two-way test (column 4). Statistically significant results (*p* < 0.05) are highlighted in bold typeface. N/A, not applicable.

**Table 2:** Endogenous *Elba2* and *His1* exhibit synthetic lethal genetic interactions.

Cross	Viability	*p-value*
+*; Tub-GAL4/TM3,Sb* × +; *pINT-H1*^*1M*^*/TM3, Sb*	17/135 (68), 25%	N/A
*Elba2*^*1*^*; Tub-GAL4/TM3,Sb* × *Elba2*^*1*^*; pINT-H1*^*1M*^*/TM3, Sb*	8/235 (118), 7%	**7.0** · **10**^**-4**^

All crosses were performed at 26 °C. In a control cross, heterozygous males that carry the *Tub-GAL4* driver on the III chromosome balanced with *TM3, Sb* were mated to females that carry *pINT-1-H1*^*1M*^ *His1* RNAi allele balanced with *TM3, Sb*. In the experimental cross, the parents additionally carried a homozygous *Elba2*^*1*^ allele on the II chromosome. Viability of the progeny was scored as the number of eclosed *Sb*+ adults relative to the total number of offspring (column 2). The expected numbers of *Sb*+ flies (calculated from the Mendelian distribution) are shown in parentheses; percent viability relative to the expected numbers is also shown. Probability value is calculated by the chi-square two-way test (column 3). *Elba2* mutation enhances the H1 depletion-dependent semilethal effect in a statistically significant fashion. N/A, not applicable.

## References

[b1] van Holde K. E. Chromatin (ed. Rich A.) (Springer-Verlag, 1989).

[b2] Wolffe A. Chromatin: Structure and Function (eds. Picknett T. & Davies S.) (Academic Press, 1998).

[b3] Ramakrishnan V. Histone H1 and chromatin higher-order structure. Crit. Rev. Eukaryot. Gene Expr. 7, 215–230 (1997). 9399071 10.1615/critreveukargeneexpr.v7.i3.20

[b4] Wolffe A. P. Histone H1. Int. J. Biochem. Cell Biol. 29, 1463–1466 (1997). 9570139 10.1016/s1357-2725(97)00026-5

[b5] Lu X. *et al.* Linker histone H1 is essential for *Drosophila* development, the establishment of pericentric heterochromatin, and a normal polytene chromosome structure. Genes Dev. 23, 452–465 (2009) 19196654 10.1101/gad.1749309 PMC2648648

[b6] Woodcock C. L., Skoultchi A. I. & Fan Y. Role of linker histone in chromatin structure and function: H1 stoichiometry and nucleosome repeat length. Chrom. Res. 14, 17–25 (2006). 16506093 10.1007/s10577-005-1024-3

[b7] Kalashnikova A. A., Rogge R. A. & Hansen J. C. Linker histone H1 and protein-protein interactions. Biochim. Biophys. Acta 1859, 455–461 (2016). 26455956 10.1016/j.bbagrm.2015.10.004 PMC4775371

[b8] Lu X. *et al.* *Drosophila* H1 regulates the genetic activity of heterochromatin by recruitment of Su(var)3-9. Science 340, 78–81 (2013). 23559249 10.1126/science.1234654 PMC3756538

[b9] Kavi H., Emelyanov A. V., Fyodorov D. V. & Skoultchi A. I. Independent biological and biochemical functions for individual structural domains of *Drosophila* linker histone H1. J. Biol. Chem. 291, 15143–15155 (2016). 27226620 10.1074/jbc.M116.730705 PMC4946930

[b10] Izzo A., Kamieniarz K. & Schneider R. The histone H1 family: specific members, specific functions? Biol. Chem. 389, 333–343 (2008). 18208346 10.1515/BC.2008.037

[b11] Fan Y. *et al.* H1 linker histones are essential for mouse development and affect nucleosome spacing *in vivo*. Mol. Cell. Biol. 23, 4559–4572 (2003). 12808097 10.1128/MCB.23.13.4559-4572.2003 PMC164858

[b12] Fu G. *et al.* Mouse oocytes and early embryos express multiple histone H1 subtypes. Biol. Reprod. 68, 1569–1576 (2003). 12606334 10.1095/biolreprod.102.012336

[b13] Perez-Montero S., Carbonell A., Moran T., Vaquero A. & Azorin F. The embryonic linker histone H1 variant of Drosophila, dBigH1, regulates zygotic genome activation. Dev. Cell 26, 578–590 (2013). 24055651 10.1016/j.devcel.2013.08.011

[b14] Abhiman S., Iyer L. M. & Aravind L. BEN: a novel domain in chromatin factors and DNA viral proteins. Bioinformatics 24, 458–461 (2008). 18203771 10.1093/bioinformatics/btn007 PMC2477736

[b15] Dai Q. *et al.* The BEN domain is a novel sequence-specific DNA-binding domain conserved in neural transcriptional repressors. Genes Dev. 27, 602–614 (2013). 23468431 10.1101/gad.213314.113 PMC3613608

[b16] Duan H. *et al.* Insensitive is a corepressor for Suppressor of Hairless and regulates Notch signalling during neural development. EMBO J. 30, 3120–3133 (2011). 21765394 10.1038/emboj.2011.218 PMC3160191

[b17] Lai E. C. Notch signaling: control of cell communication and cell fate. Development 131, 965–973 (2004). 14973298 10.1242/dev.01074

[b18] Dai Q. *et al.* BEND6 is a nuclear antagonist of Notch signaling during self-renewal of neural stem cells. Development 140, 1892–1902 (2013) 23571214 10.1242/dev.087502 PMC3631965

[b19] Dai Q. *et al.* Common and distinct DNA-binding and regulatory activities of the BEN-solo transcription factor family. Genes Dev. 29, 48–62 (2015). 25561495 10.1101/gad.252122.114 PMC4281564

[b20] Kavi H. *et al.* A genetic screen and transcript profiling reveal a shared regulatory program for *Drosophila* linker histone H1 and chromatin remodeler CHD1. G3 (Bethesda) 5, 677–687 (2015) 25628309 10.1534/g3.115.016709 PMC4390582

[b21] Aoki T., Sarkeshik A., Yates J. & Schedl P. Elba, a novel developmentally regulated chromatin boundary factor is a hetero-tripartite DNA binding complex. eLife 1, e00171 (2012). 23240086 10.7554/eLife.00171 PMC3510454

[b22] Rørth P. A modular misexpression screen in *Drosophila* detecting tissue-specific phenotypes. Proc. Natl. Acad. Sci. USA 93, 12418–12422 (1996). 8901596 10.1073/pnas.93.22.12418 PMC38006

[b23] Ner S. S. & Travers A. A. HMG-D, the *Drosophila melanogaster* homologue of HMG 1 protein, is associated with early embryonic chromatin in the absence of histone H1. EMBO J. 13, 1817–1822 (1994). 8168480 10.1002/j.1460-2075.1994.tb06450.x PMC395021

[b24] Pai C. Y., Lei E. P., Ghosh D. & Corces V. G. The centrosomal protein CP190 is a component of the *gypsy* chromatin insulator. Mol. Cell 16, 737–748 (2004). 15574329 10.1016/j.molcel.2004.11.004

[b25] Xu N., Emelyanov A. V., Fyodorov D. V. & Skoultchi A. I. *Drosophila* linker histone H1 coordinates STAT-dependent organization of heterochromatin and suppresses tumorigenesis caused by hyperactive JAK-STAT signaling. Epigenetics Chromatin 7, 16 (2014). 25177369 10.1186/1756-8935-7-16 PMC4149798

[b26] Fyodorov D. V., Blower M. D., Karpen G. H. & Kadonaga J. T. Acf1 confers unique activities to ACF/CHRAC and promotes the formation rather than disruption of chromatin *in vivo*. Genes Dev. 18, 170–183 (2004). 14752009 10.1101/gad.1139604 PMC324423

[b27] Fyodorov D. V. & Levenstein M. E. Chromatin assembly in *Drosophila* systems In Current Protocols in Molecular Biology (ed. Chanda V. B.) 21.27.21–21.27.27 (Wiley & Sons, 2002). 18265309 10.1002/0471142727.mb2107s58

[b28] Konev A. Y. *et al.* CHD1 motor protein is required for deposition of histone variant H3.3 into chromatin *in vivo*. Science 317, 1087–1090 (2007). 17717186 10.1126/science.1145339 PMC3014568

[b29] Foe V. E. & Alberts B. M. Studies of nuclear and cytoplasmic behaviour during the five mitotic cycles that precede gastrulation in *Drosophila* embryogenesis. J. Cell Sci. 61, 31–70 (1983). 6411748 10.1242/jcs.61.1.31

