# Supplementary material for: BEN domain protein Elba2 can functionally substitute for linker histone H1 in Drosophila in vivo
Source: Sci Rep. 2016 Sep 30;6:34354. doi: 10.1038/srep34354 (PMC5043383; doi:10.1038/srep34354)
Supplement: Supplementary Information [file srep34354-s1.pdf]

BEN domain protein Elba2 can functionally substitute for linker histone H1 in *Drosophila* in vivo

Na Xu[1,+], Xingwu Lu[2,+], Harsh Kavi[3,+], Alexander V. Emelyanov, Travis J. Bernardo, Elena Vershilova, Arthur I. Skoultchi*, and Dmitry V. Fyodorov*

SUPPLEMENT

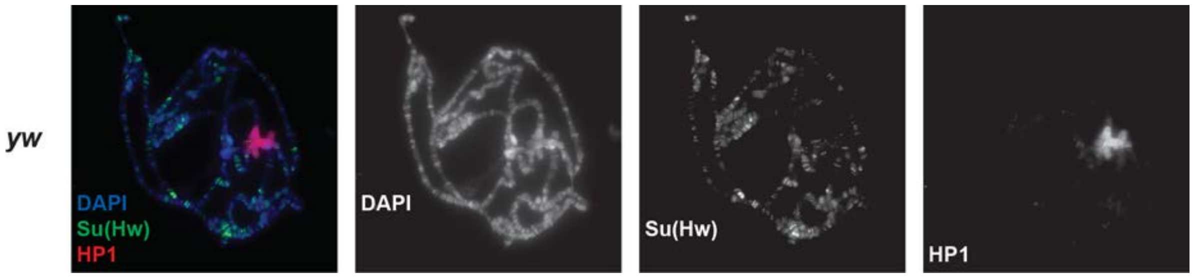

Figure S1. Su(Hw) distribution in polytene chromosomes.

Endogenous Su(Hw) protein is not ubiquitously distributed in polytene chromosome. Instead, it Su(Hw) is restricted to discrete loci in euchromatic arms and heterochromatin. Polytene chromosomes of salivary glands from wild-type (*yw*) larvae were stained with DAPI (blue) as well as with Su(Hw) (green) and HP1 (red) antibodies.

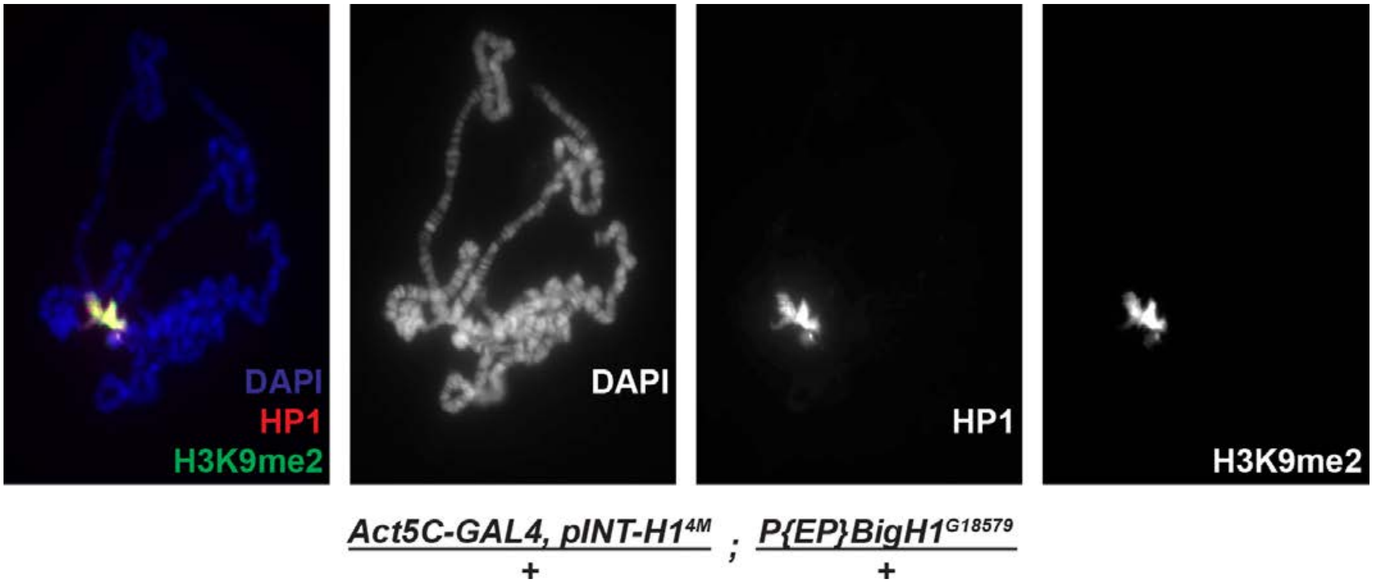

Figure S2. The rescue of H1 depletion phenotypes by ectopic expression of BigH1.

Polytene chromosome defects due to H1 depletion in vivo are ameliorated by ectopic mis-expression of *BigH1*. Polytene chromosomes prepared from L3 larvae depleted of H1 and ectopically overexpressing BigH1 were stained with DAPI (blue) and antibodies against HP1 (red) and H3K9me2 (green) as in **Fig. 3b**. The appearance of H1-depleted polytene chromosomes in the *Act5C-GAL4, pINT-H1*^{4M}/+ background is shown in Fig. 3a and 3b (middle panels).

Inter se crosses	Homozygote viability	*p*-value	Female/male fertile
+/CyO (*inter se wt*) precise excision	72/225 (75), 96%	N/A	Y/Y
Elba2^{1}*/CyO* (*inter se Elba2*^{1}) Δ1,204 bp	52/178 (59), 88%	0.55	Y/Y
Elba2^{2}*/CyO* (*inter se Elba2*^{2}) Δ961 bp	44/145 (48), 91%	0.74	Y/Y
Elba2^{3}*/CyO* (*inter se Elba2*^{3}) Δ960 bp	80/253 (84), 95%	0.93	Y/Y
Elba2^{4}*/CyO* (*inter se Elba2*^{4}) Δ783 bp	101/311 (104), 97%	0.91	Y/Y

Table S1. *Drosophila Elba2* is not essential. All crosses were performed at 26°C. Heterozygous *Elba2* parents (**Fig. S1**) balanced with *CyO* were mated *inter se*, and viability of the progeny was scored as the number of eclosed *Cy+* adults relative to the total number of offspring (column 2). Expected numbers of *Cy+* flies (calculated from the Mendelian distribution) are shown in parentheses; percent viability relative to the expected numbers is also shown. In an isogenic control cross (row 2), heterozygous parents that carry a precise excision of *P{EP}Elba2*^{G17999} were used. Probability values are calculated by the chi-square two-way test (column 3). Homozygous progeny were also tested for male and female sterility (column 4). *Elba2* mutation does not significantly affect adult viability or fertility. N/A, not applicable; Y, yes.
